# Patterns of Retinal Ganglion Cell Damage in Neurodegenerative Disorders: Parvocellular vs Magnocellular Degeneration in Optical Coherence Tomography Studies

**DOI:** 10.3389/fneur.2017.00710

**Published:** 2017-12-22

**Authors:** Chiara La Morgia, Lidia Di Vito, Valerio Carelli, Michele Carbonelli

**Affiliations:** ^1^IRCCS Institute of Neurological Sciences of Bologna, Bellaria Hospital, Bologna, Italy; ^2^Neurology Unit, Department of Biomedical and Neuromotor Sciences, University of Bologna, Bologna, Italy

**Keywords:** optic nerve, retinal ganglion cells, optical coherence tomography, Parkinson’s disease, Alzheimer’s disease, Huntington’s disease, glaucoma, multiple system atrophy

## Abstract

Many neurodegenerative disorders, such as Parkinson’s disease (PD) and Alzheimer’s disease (AD), are characterized by loss of retinal ganglion cells (RGCs) as part of the neurodegenerative process. Optical coherence tomography (OCT) studies demonstrated variable degree of optic atrophy in these diseases. However, the pattern of degenerative changes affecting the optic nerve (ON) can be different. In particular, neurodegeneration is more evident for magnocellular RGCs in AD and multiple system atrophy with a pattern resembling glaucoma. Conversely, in PD and Huntington’s disease, the parvocellular RGCs are more vulnerable. This latter pattern closely resembles that of mitochondrial optic neuropathies, possibly pointing to similar pathogenic mechanisms. In this review, the currently available evidences on OCT findings in these neurodegenerative disorders are summarized with particular emphasis on the different pattern of RGC loss. The ON degeneration could become a validated biomarker of the disease, which may turn useful to follow natural history and possibly assess therapeutic efficacy.

## Introduction

Many neurodegenerative disorders are characterized by increasing evidences that optic nerve (ON) degeneration is part of the central nervous system neurodegenerative process. In fact, optical coherence tomography (OCT) and histological postmortem studies documented loss of retinal ganglion cells (RGCs) and their ON-forming axons in neurodegenerative disorders such as Alzheimer’s disease (AD) (1–4), Parkinson’s disease (PD) (5), Huntington’s disease (HD) (6), multiple system atrophy (MSA) (7, 8), spinocerebellar ataxias (9), spastic paraparesis (10), and others.

Retinal ganglion cells are neurons located in the retinal ganglion cell layer (GCL) characterized by a soma from which the originating axon runs initially in the retinal nerve fiber layer (RNFL). Then, these axons converge turning into the optic disc, cross the lamina cribrosa at the optic nerve head (ONH), and constitute the ON. They are particularly sensitive to neurodegenerative damage due to defective mitochondrial dynamics and axonal transport, as well as oxidative stress and energy depletion, given the high metabolic demand and performances typical of these cells, mostly determined by the asymmetric myelination (11, 12).

Moreover, the deposition in RGCs of α-synuclein and β-amyloid (Aβ), typical hallmarks of these neurodegenerative disorders, has been already documented, respectively, in PD (13, 14) and AD (2, 15, 16).

Interestingly, the pattern of RGC loss described in these neurodegenerative disorders can be different. In fact, the axonal damage in AD is described as typically affecting the magnocellular cells (M-cells), which is reflected by a preferential RNFL thinning in the superior and inferior quadrants (17). This pattern of RGC loss, which is also reported in MSA (7, 8), resembles that described in glaucoma, where a preferential loss of M-cells is established (18).

Conversely, RGC loss in PD involves preferentially the parvocellular cells (P-cells), which is reflected by the temporal thinning of the RNFL (17). This pattern is similar to what is described for mitochondrial optic neuropathies, where typically the pathology affects the papillomacular bundle (PMB) and is hallmarked by the preferential loss of the P-cells leading to temporal pallor of the optic disc and a central visual field defect (11, 19).

This review is aimed at critically highlighting the differential pattern of RGC loss in some paradigmatic neurodegenerative disorders such as AD and PD, based on the main OCT and histological findings derived by literature.

## RGC Features

Visual information generated by photoreceptors is transmitted through bipolar cells to RGCs, whose axons leave the eye reaching the lateral geniculate nucleus (LGN). At least 30 different morphological types of RGCs have been recognized in the human retina, but only some of them are judged essential for the function of the four different visual pathways (20).

The midget RGCs (about 80% of total RGCs in the monkey retina) project to the parvocellular layers of the LGN, the parasol RGCs (5–15%) project to the magnocellular layers of the LGN, the bistratified RGCs form part of the koniocellular visual pathway projecting to the koniocellular layers of the LGN, and finally the melanopsin-containing RGCs (mRGCs, about 1% of total RGCs) project mostly to the suprachiasmatic nucleus of the hypothalamus, constituting the retino-hypothalamic pathway.

RGCs are characterized by specific common features, such as the location of their somata in the GCL, the arborization of their dendrites in the inner plexiform layer (IPL), and the gathering of their axons in the ON, which, by projecting to the LGN, connect their glutamatergic synapsis with the second order neurons of the visual pathway (21).

An important characteristic of the P-pathway is the existence of very small midget cells (or P-cells) that are connected to single-cone midget bipolar cells in the central 2 mm around the fovea (22, 23). This one-to-one neuronal circuitry is thought to form the basis of red–green color opponency. The parasol cells (or M-cells) constitute a smaller proportion of RGCs and their long (L) and middle (M) wavelength-sensitive cone inputs form the magnocellular pathway, which transmits achromatic visual information of high temporal but low spatial resolution and luminance and movement messages (24). The M-cells of primates have peculiar features represented by large cell bodies, thick axons, and wide radial branching dendritic trees. At difference, P-cells have small cell bodies, thin axons, and narrow dendritic trees with a more bushy and dense branching pattern (25). Moreover, large fibers exhibit faster pulse conduction than the smaller fibers (26).

Both RGC classes increase in size with increasing distance from the foveal slope, maintaining their distinct branching pattern at all eccentricities, but the average P-cell dendritic-field diameter is smaller than mean M-cell dendritic field throughout the retina.

At the ONH, RGC axons are distributed in a specific topographic manner (27). In fact, the retinal nerve fibers from the nasal half of the retina step directly into the optic disc as superior and inferior radiating fibers, fibers from the macular area come horizontally as PMB, and fibers from the temporal retina arch above and below the macula as superior and inferior arcuate fibers (27).

**Figure 1:**
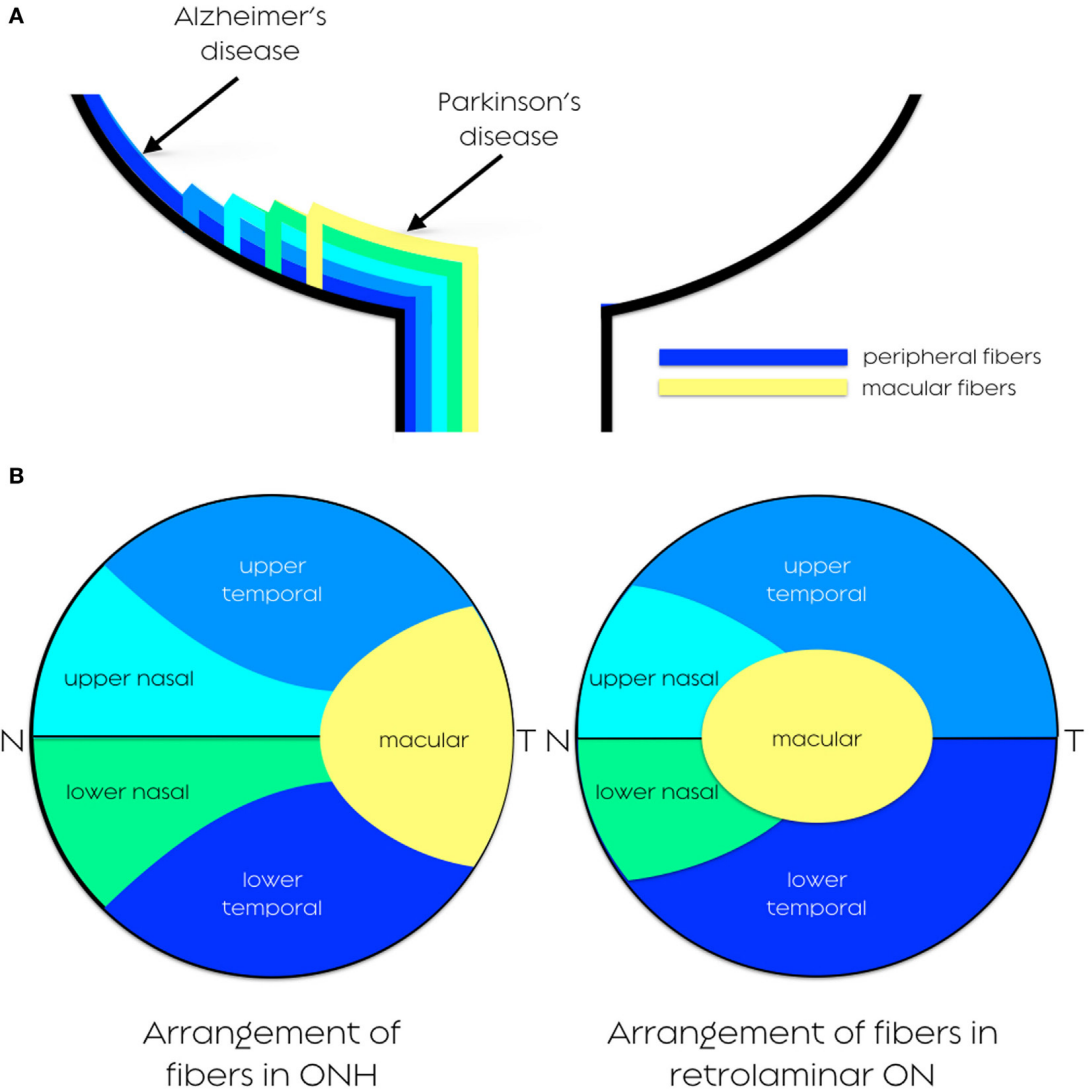
**(A)** Distribution of retinal nerve fibers. Cross-sectional arrangement of axons, with deeper fibers originating from peripheral retina running closer to choroid occupying peripheral location in ONH, and superficial fibers originating nearer to the ONH occupy a more central portion of the nerve. **(B)** Arrangement of nerve fibers in the anterior visual pathway. In the pre-laminar, laminar, and proximal retro-laminar region of the ON, the axons from PMB are arranged temporally in the nerve but along the path to the LGN, they gradually shift centrally. ON, optic nerve; ONH, optic nerve head; PMB, papillomacular bundle; LGN, lateral geniculate nucleus.

The above described anatomy of the P-pathway (one-to-one connection with bipolar cells in the central 2 mm around the fovea and the conveyance of color information) implies that the P-cells are predominantly represented by PMB entering the ONH from the temporal quadrant. Furthermore, topographically, deeper fibers from peripheral retina occupy peripheral location in the ONH (neighboring to the ONH edge), and superficial fibers from central retina occupy central location in the ONH (Figure 1A). Therefore, in the retro-laminar region of the ON, the axons from PMB (predominantly formed by p-RGCs) are arranged temporally in the nerve but along the path to the LGN, they gradually shift centrally, whereas larger fibers (predominantly formed by p-RGCs) are located in greater proportion in the periphery of the nerve (28) (Figure 1B).

## OCT Findings in Neurodegenerative Diseases with Preferential Parvocellular Damage

### Parkinson’s Disease

Parkinson’s disease is a neurodegenerative disease whose core clinical features are bradykinesia, resting tremor or rigidity, and response to l-DOPA therapy (29). PD prevalence increases with age, which is the single most relevant risk factor (30). Visual disturbances described in PD may range from reduced visual acuity and color vision to abnormal contrast sensitivity, dysfunction of eye movements and visual hallucinations (31, 32).

Moreover, the occurrence of ON pathology is reported by numerous OCT studies including time-domain and spectral-domain studies (33–56) (Table 1).

**Table 1:** OCT results in PD.

	OCT	No. of patients	Results
Inzelberg et al. (33)	Zeiss 3000 Stratus	10 PD 10 controls	↓ avg; inf-temp RNFL thickness
Yavas et al. (34)	Heidelberg Retinal Tomography	44 PD 21 controls	↓ avg; nasal, sup and inf-nas, and inf-temp RNFL thickness
Altintas˛ et al. (35)	Zeiss 3000 Stratus	17 PD 11 controls	↓ avg; sup and nas RNFL thickness
Moschos et al. (38)	Zeiss 3000 Stratus	16 PD 20 controls	↓ inf and temp RNFL thickness
Garcia-Martin et al. (39)	Cirrus and Spectralis	75 PD 75 controls	↓ RNFL thickness in all four quadrants
Rohani et al. (40)	Topcon 3D OCT	27 PD 27 controls	↓ avg; all quadrants RNFL thickness
Kirbas et al. (41)	Cirrus	42 PD 40 controls	↓ avg; temp RNFL thickness
La Morgia et al. (42)	Zeiss 3000 Stratus	43 PD 86 controls	↓ temp RNFL thickness
Satue et al. (43)	Cirrus and Spectralis	100 PD 100 controls	↓ inf (Cirrus and Spectralis) RNFL thickness
Sen et al. (45)	Not mentioned in the abstract	35 PD 11 controls	↓ avg RNFL thickness
Satue et al. (47)	Cirrus and Spectralis	153 PD 242 controls	↓ avg (Cirrus and Spectralis); sup, inf, and temp (Cirrus); sup and inf (Spectralis) RNFL thickness
Garcia-Martin et al. (48)	Cirrus and Spectralis	46 PD 33 control	↓ avg (Cirrus and Spectralis); sup, inf, and temp (Cirrus); sup and temp (Spectralis) RNFL thickness
Garcia-Martin et al. (49)	Spectralis	129 PD 129 controls	↓ avg; nas-inf, temp-inf, and temp-sup RNFL thickness
Jiménez et al. (50)	Zeiss 3000 Stratus	52 PD 50 controls	↓ avg; all four quadrants RNFL thickness
Bayhan et al. (51)	SD-OCT (RTVue-100)	20 PD 30 controls	↓ nas RNFL thickness
Sari et al. (52)	SD-OCT	54 PD 54 controls	↓ inf and temp RNFL thickness
Kaur et al. (53)	SD-OCT	20 PD 20 controls	↓ avg; sup and temp RNFL thickness
Eraslan et al. (55)	SD-OCT (RTVue-100)	25 PD 23 controls	↓ avg RNFL thickness
Pilat et al. (56)	SD-OCT (Copernicus)	25 PD 25 controls	↓ avg; all four quadrants RNFL thickness

*PD, Parkinson’s disease; avg, average; temp, temporal; sup, superior; inf, inferior; nas, nasal; RNFL, retinal nerve fiber layer; SD, spectral domain; OCT, optical coherence tomography*.

Interestingly, the pattern of axonal loss reported by OCT studies points toward a preferential involvement of the temporal quadrant (5, 33, 38, 41–43, 48, 49, 52, 53, 57). Moreover, the evidence of a more severe RGC loss in the eye contralateral to the most affected body side is suggestive of a congruently asymmetric degenerative process in PD (42). This pattern of axonal loss is clearly distinguishable from the preferential loss of M-cells documented in other parkinsonian syndromes such as MSA (7, 8) and resembles mitochondrial optic neuropathies (11). The most common mitochondrial optic neuropathies, i.e., Leber’s hereditary optic neuropathies (LHON) and dominant optic atrophy (DOA) are, in fact, typically characterized by ON pathology involving preferentially the smallest axons constituting the PMB leading to temporal pallor and central scotoma (11, 19). The P-cells are energetically more vulnerable to mitochondrial dysfunction, as clearly described by recent histological studies and mathematical modeling of the RGC loss in LHON (58). This susceptibility to mitochondrial dysfunction and particularly to oxidative stress may be related to the high energetic demand in relation to a low energetic potential, which is due to the high surface area/volume ratio, and to the absence of myelin around the axons in the unmyelinated portion of the ON (58).

In this context, the similar pattern of axonal loss in the ON characterizing PD and mitochondrial optic neuropathies has a common ground in the documented complex I defect and, more in general, mitochondrial dysfunction reported in PD (59).

Moreover, in PD eyes, it has been demonstrated that the area around the fovea, where INL and GCL emerge, is characterized by a significant remodeling as consequence of the neurodegeneration involving the inner nuclear layers and has been indicated as a marker of the disease with the best discriminative power compared with controls (60). Interestingly, as for the RNFL results reported by OCT studies (42), it has been described an interocular asymmetry for the foveal thickness, pointing again to an asymmetry of the neurodegenerative process in PD, congruent with the motor symptoms of the disease (61). Finally, recent studies demonstrated the presence of α-synuclein deposition in the retina of PD patients. This deposition is particularly evident in the inner retina including the GCL, the IPL, and the interface between IPL and inner nuclear layer (13, 14). Interestingly, there is a documented direct relationship between intracellular deposition of α-synuclein and mitochondrial dysfunction (59).

### Huntington’s Disease

Huntington’s disease is a neurodegenerative disease inherited as an autosomal dominant trait, whose main features are the occurrence of choreic movements, as well as psychiatric and cognitive disturbances in young-adult individuals (62).

Moreover, HD patients early in the disease course suffer sleep and circadian dysfunction, which contribute to the cognitive deterioration (63). The presence of retinal degeneration, and in particular of RGC loss, has been recently reported by OCT studies (6, 64). In particular, Kersten and coauthors demonstrated that in these patients there is a RNFL thinning in the temporal sector correlating with the disease duration, similar to PD (64). As for PD, this axonal loss pattern may be explained by the role attributed to mitochondrial dysfunction in the pathogenesis of the disease. In particular, recent experimental evidences in HD point to an abnormal mitochondrial dynamics with defective fission (65, 66).

## OCT Findings in Neurodegenerative Diseases with Preferential Magnocellular Damage

### Alzheimer’s Disease

Alzheimer’s disease is the most frequent cause of dementia and is hallmarked by the accumulation of amyloid plaques and neurofibrillary tangles in the brain (67). AD is characterized by visual disturbances occurring early in the course of the disease and reflecting neuronal damage of the cerebral visual pathway. The symptoms affect various aspects of visual function such as visual field, color vision, contrast sensitivity, motion perception, visuospatial construction, visual attention, and fixation (68).

Several histological studies in AD demonstrated the impairment of the entire visual pathway, documented initially in the brain and subsequently in the retina and ON (69–75). In 1986, Hinton and colleagues provided the first evidences of optic neuropathy in AD, describing loss of RGCs and axons at postmortem histology of the ON (1). Subsequently, other histological studies showed degeneration of the inner retina, more pronounced in the superior and inferior quadrant of the ON (2, 70–75). In 2011, Koronyo-Hamaoui and colleagues documented for the first time extracerebral Aβ deposits in postmortem human retinas of AD patients and *ex vivo* in APPSWE/PS1ΔE9 transgenic mice after curcumin administration (15). In recent years, other histological studies of human retina confirmed the occurrence of extracellular plaques and intracellular Aβ deposits in the inner retinal layers involving mainly the superior hemiretina (2, 16, 76, 77). La Morgia and colleagues demonstrated that mRGCs, a subgroup of RGCs intrinsically photosensitive, are selectively affected by the amyloid pathology in AD. Remarkably, the loss of these cells occurred even with a normal RGC count, pointing to a specific AD pathology affecting mRGCs (2).

With the advent, in the last 15 years, of OCT, a non-invasive optical imaging technique of the retina and ONH, many studies investigated the occurrence of ON pathology in AD and five comprehensive meta-analyses summarized the results provided by these OCT studies (3, 4, 78–80) (Table 2).

**Table 2:** OCT results in AD.

	OCT	No. of patients			Results
		AD	MCI	HC	
Pillai et al. (82)	Cirrus 4000 HD	21	20	34	No significant differences
La Morgia et al. (2)	Stratus OCT	21		74	↓ avg; sup RNFL thickness
Salobrar-Garcia et al. (83)	OCT1000 Topcon	23		28	No significant differences
Eraslan et al. (84)	RTVue-100	18		20	↓ RNFL thickness and macular GCC thickness
Günes˛ et al. (85)	OPKO/OTI SD-OCT	20		20	↓ avg RNFL thickness
Liu et al. (86)	Stratus OCT	67	26	39	↓ avg; sup RNFL thickness
Oktem et al. (87)	Cirrus HDOCT	35	35	35	↓ avg RNFL thickness
Cheung et al. (88)	Cirrus HDOCT	100	41	123	↓ GC-IPL thickness in all macular sectors↓ Superior RNFL thickness
Gao et al. (89)	Cirrus HDOCT	25	26	21	↓ avg; sup RNFL thickness↓ Macular volume in AD and MCI
Bambo et al. (90)	Cirrus HDOCT	56		56	↓ avg RNFL thickness
Larrosa et al. (91)	Cirrus HDOCT	151		61	↓ avg; sup and inf RNFL thickness
Ascaso et al. (92)	Stratus OCT	18	21	41	↓ avg RNFL thickness↓ Macular volume in MCI vs HC
Polo et al. (93)	Cirrus HDOCT Heidelberg Spectralis	70		70	↓ avg; sup and inf RNFL thickness
Gharbiya et al. (94)	Heidelberg Spectralis	21		21	No differences in RNFL thickness ↓ Choroidal thickness
Kromer et al. (95)	Heidelberg Spectralis	22		22	↓ nas and sup sectors RNFL thickness
Moreno-Ramos et al. (96)	OCT1000 Topcon	10		10	↓ avg RNFL thickness
Marziani et al. (97)	RTVue-100 Heidelberg Spectralis	21		21	↓ Macular RNFL and macular RNFL + GCL in all sectors
Kirbas et al. (98)	OCT1000 Topcon	40		40	↓ avg; sup RNFL thickness
Moschos et al. (99)	Stratus OCT	30		30	↓ avg; sup and inf RNFL thickness
Kesler et al. (100)	Stratus OCT	30	24	24	↓ avg; sup (AD) and inf (MCI) RNFL thickness
Lu et al. (101)	Stratus OCT	22		22	↓ avg; sup and inf RNFL thickness
Paquet et al. (102)	Stratus OCT	26	23	15	↓ avg RNFL thickness
Berisha et al. (103)	Stratus OCT	9		8	↓ RNFL thickness in the superior quadrant
Iseri et al. (104)	Stratus OCT	14		15	↓ avg RNFL thickness↓ Macular volume
Parisi et al. (105)	Stratus OCT	17		14	↓ avg RNFL thickness

*AD, Alzheimer’s disease; avg, average; temp, temporal; sup, superior; inf, inferior; nas, nasal; RNFL, retinal nerve fiber layer; GCC, ganglion cell complex; GCL, ganglion cell layer; OCT, optical coherence tomography; IPL, inner plexiform layer; MCI, mild cognitive impairment*.

In 2017, the most recent meta-analysis by den Haan and colleagues described the results on the average peripapillary RNFL in 24 studies including 887 AD patients and 864 controls, and the 4 peripapillary RNFL quadrants in 20 studies. The RNFL thickness was thinner in AD compared with controls [standardized mean difference (SMD) −0.98], corresponding to an absolute reduction of about 10 µm. RNFL thinning was more pronounced in the superior and inferior peripapillary quadrants and was age-related (Table 2) (3). The same meta-analysis reported also data about mild cognitive impairment (MCI) patients (322 AD patients, 216 MCI patients, and 367 healthy controls), and RNFL thickness of MCI patients resulted intermediate between AD patients and healthy controls with a SMD of −0.71 compared with controls.

Furthermore, seven studies reported that total macular thickness is significantly reduced in AD retinas compared with controls with the largest effect on the outer macular ring [according to standard macular measures from the Early Treatment Diabetic Retinopathy Study (ETDRS)] (81). Moreover, the meta-regression by den Haan showed that OCT type, mini-mental state examination score, glaucoma exclusion score, and age were not associated with the SMD in the AD group compared with controls.

Overall, it must be emphasized that the results of OCT studies in AD are quite heterogeneous, due to the relatively small sample sizes and the different methods used for the data analysis. Moreover, not all the studies examined reported a thinning of the RNFL in AD patients (82). The main results of OCT studies in AD are summarized in Table 2 (82–105). Notwithstanding the technical limitations and some contrasting results, a specific pattern of axonal loss clearly emerged in the ON of AD patients, closely resembling the pattern of RGC loss described in glaucoma, i.e., the RNFL atrophy in the superior and inferior quadrants (18).

The relative sparing of the RNFL in the temporal quadrant and the predominant involvement of the superior and inferior RNFL quadrants (e.g., SDM for AD vs controls in temporal sector was −0.42 vs −0.99 in superior sector) indicate a preferential contribution of parasol RGCs projecting to the magnocellular pathway (M-cells), which are mainly located in the extra-macular retina and are not specifically contributing to visual acuity (16, 24). In this context, it should be mentioned that some authors suggested that RNFL thinning in the superior and inferior quadrants might be justified by the fact that more neurons physiologically are located in these quadrants where therefore neurodegeneration is more apparent (equal percentage corresponds to a greater absolute reduction in thickness) (3). However, this pattern remains clearly distinguishable from that described in PD, where a predominant loss of the P-cells is reported (57). Furthermore, recent histological findings in postmortem AD retinal specimens reported that the axonal loss predominantly affected the larger fibers in the superior quadrants, and, to a lesser extent, the nasal and inferior quadrants, whereas the temporal quadrants, were largely spared (2, 16). The reasons why the M-cells are more vulnerable to AD pathology is still unknown but might be related to the different vulnerability of RGCs to amyloid deposition, which has been already demonstrated in mRGCs, characterized by a big soma and branched dendrites, similarly to M-cells (2).

### Multiple System Atrophy

Multiple system atrophy is a neurodegenerative disorder typically defined by parkinsonian and cerebellar features and autonomic failure (106). The occurrence of RGC loss has been recently reported by different OCT studies (8).

Mendoza-Santiesteban and colleagues compared 24 MSA patients to 20 PD and 35 controls demonstrating a significant RNFL and ganglion cell complex (GCL + IPL) thinning in the MSA patients compared with controls. Interestingly, in the MSA group the RNFL thinning was significant in the inferior quadrant relatively sparing the temporal region, thus clearly distinguishable from the PD cohort where a predominant temporal pattern was consistently found. The authors speculated that the different pattern of axonal loss could be due to different patterns of myelination by oligodendrocytes of M-cells axons (7), which might also explain the absence of visual complaints and normal visual acuity reported by MSA patients (7, 8). Similar results in terms of pattern of RNFL loss have been reported also by other studies (107–109).

### Glaucoma

Elevated intraocular pressure (IOP) is the main risk factor for glaucoma, but even glaucomatous patients with IOP within normal limit will progress in loosing RGCs (110). The hypothesis that larger RGCs are preferentially affected in human, and experimental glaucoma has received remarkable credit since Quigley and colleagues formulated it in 1987 (18, 28, 111). This hypothesis was corroborated by postmortem examination of the human LGN of glaucoma patients where a selective neuronal loss in the layers receiving input from parasol cells was shown (112). Over the years, the selective damage of M-cells or S-cone pathway (113) has been debated with contrasting opinions (114–117).

Nonetheless, recent studies in experimental glaucoma demonstrated that RGCs undergo morphologic changes before cell death, which are represented by reduction of soma volume, axon size, and dendritic tree area. These changes are consistent with cell shrinkage as an explanation for the apparent survival of midget cells reported in earlier studies (118). Weber and colleagues (119, 120) found a reduction in thickness and complexity of the dendritic tree in primate glaucomatous retinas, highlighting that M-cells and P-cells were involved to a similar extent (121). Moreover, psychophysical studies comparing responses of M and P pathways, found contrasting results, some supporting similar dysfunction for both pathways (122), whereas others suggested that visual functions such as contrast sensitivity and contrast gain signature, mediated by the M pathway, were reduced in glaucoma (123).

Even if the mechanisms underlying axonal damage in glaucoma are still not completely understood, the pattern of peripapillar and macular RNFL thinning is now well described by OCT studies.

Schuman and colleagues in 1995 (124) showed for the first time a thinning of the RNFL in glaucomatous eyes as compared with normal eyes, more evident in the inferior quadrant. In 2005, using Stratus OCT (time-domain OCT) Leung and colleagues noticed the greatest reductions in peripapillary RNFL thickness in glaucoma at the superotemporal (11 o’clock) and inferotemporal (7 o’clock) sectors. These changes are congruent with the distribution of the most commonly reported visual field defect in glaucoma (125). Subsequently, numerous OCT studies have confirmed the typical pattern of relative sparing of peripapillary RNFL in the temporal quadrant and the resulting greater diagnostic performance to distinguish normal eyes from eyes with early glaucoma by looking at the inferior (inferotemporal) and superior (superotemporal) RNFL quadrants (126). In more recent years, with the advent of spectral-domain OCT and its greater spatial resolution, it has been possible to measure with higher reliability the thickness of the RNFL and the retinal ganglion cell plus inner plexiform (RGC+) just in the macula (127). Hood and coauthors demonstrated a greater thinning of the macular RGC+ thickness in the inferior macula (superior visual field) in glaucoma patients (128), corresponding to the typical arcuate RGC damage associated with local peripapillar RNFL thinning in a confined region of the disc, which the authors named “the macular vulnerability zone” (about 7:00 o’clock). Furthermore, the authors reported that the temporal region of the disc, where axons come from the upper and nasal macula, showed a milder damage until late stages of the disease.

Overall, the pattern described by OCT studies in glaucoma clearly points to a predominant damage of the inferior and superior ON quadrants where M-cells are preferentially located, with a relative sparing of the temporal sector (P-cells), similar to what has been described in AD and MSA.

### Conclusions

Optical coherence tomography is an extraordinary tool to assess anatomy *in vivo*, to describe subtle differences in the patterns of neurodegeneration and to provide possible mechanistic insights for ON damage in different human diseases.

Mechanisms of neurodegeneration may act at different levels of the RGC/ON system, which is composed by RGC dendritic tree, the soma, the axon in its unmyelinated intraretinal component and the transition through the lamina cribrosa at the ONH, and the post-laminar myelinated component. Moreover, large and small RGCs and thicker and thinner axons display different conduction velocities, thus metabolic requirements and myelin sheath turnover. All these elements, and others that we know less, such as vascularization, support from glial cells and anatomical microenvironment come into play, possibly differentiating mechanistically the neurodegenerative pattern.

It must be also considered that a trans-synaptic degeneration may occur in some circumstances, such as in the specific case documented in AD where both the SCN and the mRGCs are affected by amyloid deposition supporting the hypothesis of a global involvement of the retino-hypothalamic tract (2, 129). Despite evidences are not conclusive it cannot be excluded that retinal degeneration in these disorders can be also contributed by trans-synaptic neurodegeneration.

Current evidences for PD suggest a number of possible co-occurring pathological events. Dopaminergic depletion may affect the connecting circuitry such as the amacrine cells, possibly leading to RGC de-afferentation and dendritic remodeling. However, mounting evidences highlight α-synuclein deposition in PD retinas, and mitochondrial dysfunction, not surprisingly, may ultimately lead to a prevalent damage of the P-cells. This is consistent with current OCT results pointing to a predominant “mitochondrial-like” pattern of ON degeneration. The α-synuclein deposition in the retina, which might support a primary neurodegenerative process, parallels the recent findings of α-synuclein deposition demonstrated in the skin nerves of PD patients (130).

In AD, similar to PD, there is deposition of an abnormally folded protein, i.e., β-amyloid, which, however, presents with a peculiar retinal topography involving preferentially the peripheral retina in the superior quadrant, as recently shown (16). At this regard, Koronyo and coauthors have recently demonstrated that amyloid plaques can be visualized *in vivo* in AD human eyes using oral curcumin, opening the possibility to use the eye as a reliable and easily accessible biomarker for this disease (16).

Overall, in AD the OCT results, as well as the histological studies, point to a predominant affection of the M-cells, which somehow links AD to what is observed in glaucoma. This parallel may suggest that a relevant role is played by the ONH anatomy, where fibers turn 90° to engage into the transition through the lamina cribrosa, where a still poorly understood mechanism hits the larger axons driving M-RGC loss. The dichotomy between the two opposite patterns of prevalent P-cells vs M-cells vulnerability is further reflected in other two neurodegenerative disorders, HD as opposed to MSA. Remarkably, MSA that is a synucleinopathy with parkinsonism displays an OCT pattern more similar to AD, whereas HD is closer to PD. This latter link may be again supported by the common intrinsic mitochondrial dysfunction, whereas the link between MSA and AD in terms of retinal pathology remains puzzling and deserves further investigations.

Overall, OCT proves to be a powerful tool to assess anatomically neurodegeneration *in vivo* providing, once solidly validated by complementary postmortem histological studies, a great potential in all neurodegenerative disorders for monitoring natural history and ultimately possibly validate neuroprotective therapeutic strategies by proving their efficacy.

## Author Contributions

CLM and MC were responsible for conception, design, drafting, and revision of the manuscript. LV was responsible for drafting and revision of the manuscript. VC was responsible for conception and revision of the manuscript.

## Conflict of Interest Statement

The authors declare that the research was conducted in the absence of any commercial or financial relationships that could be construed as a potential conflict of interest.
